# Bayesian Generalized Linear Model for Simulating Bacterial Inactivation/Growth Considering Variability and Uncertainty

**DOI:** 10.3389/fmicb.2021.674364

**Published:** 2021-06-24

**Authors:** Satoko Hiura, Hiroki Abe, Kento Koyama, Shige Koseki

**Affiliations:** Graduate School of Agricultural Science, Hokkaido University, Sapporo, Japan

**Keywords:** parameter estimation, Bayesian inference, generalized linear model, poisson distribution, negative binomial distribution, model residual

## Abstract

Conventional regression analysis using the least-squares method has been applied to describe bacterial behavior logarithmically. However, only the normal distribution is used as the error distribution in the least-squares method, and the variability and uncertainty related to bacterial behavior are not considered. In this paper, we propose Bayesian statistical modeling based on a generalized linear model (GLM) that considers variability and uncertainty while fitting the model to colony count data. We investigated the inactivation kinetic data of *Bacillus simplex* with an initial cell count of 10^5^ and the growth kinetic data of *Listeria monocytogenes* with an initial cell count of 10^4^. The residual of the GLM was described using a Poisson distribution for the initial cell number and inactivation process and using a negative binomial distribution for the cell number variation during growth. The model parameters could be obtained considering the uncertainty by Bayesian inference. The Bayesian GLM successfully described the results of over 50 replications of bacterial inactivation with average of initial cell numbers of 10^1^, 10^2^, and 10^3^ and growth with average of initial cell numbers of 10^–1^, 10^0^, and 10^1^. The accuracy of the developed model revealed that more than 90% of the observed cell numbers except for growth with initial cell numbers of 10^1^ were within the 95% prediction interval. In addition, parameter uncertainty could be expressed as an arbitrary probability distribution. The analysis procedures can be consistently applied to the simulation process through fitting. The Bayesian inference method based on the GLM clearly explains the variability and uncertainty in bacterial population behavior, which can serve as useful information for risk assessment related to food borne pathogens.

## Introduction

Predictive microbiology models explain bacterial number variations over time and how growth/inactivation rates are affected by environmental conditions (Lammerding and Fazil, 2000). In the development process of mathematical or statistical models, experimental data are collected, a model is selected, and curve fitting is applied to the data for parameter estimation. Least-squares estimation has been the most widely used curve fitting procedure (Gil et al., 2017). The least-squares methods in frequentist statistics assume that the experimental error follows a normal distribution, and studies conducted thus far have described the experimental error using a normal distribution (van Boekel, 2020). In the case of bacterial growth or inactivation kinetics, the model residual with respect the logarithmic number of cells has been assumed to follow a normal distribution (Ratkowsky et al., 1996), though the reason for this assumption is unclear given the variability and uncertainty in bacterial population behavior. The current model residual based on the normal distribution cannot clarify the origin of the error, which means that we need to identify what type of error is included, how the error can be separated, and how large the error is.

A predictive model has been employed for exposure assessment in risk assessment to quantify the changes in the number of bacteria along the farm-to-fork chain. Exposure assessment is necessary to qualitatively and/or quantitatively assess the likelihood of ingestion of pathogens (FAO/WHO, 2008). A quantitative exposure assessment requires the development of a model that mathematically describes all the relationships between the factors influencing the exposure (FAO/WHO, 2008). Since point estimation is performed using the mean values in the kinetic model (FAO/WHO, 2008), it is difficult to appropriately estimate the changes in bacterial behavior characterized by individual cell variation. To describe the variation in bacterial behavior considering the variability and uncertainty, the need to distinguish between variability and uncertainty has been pointed out (Nauta, 2000).

The generalized linear model (GLM) is an approach incorporating various probability distributions into a fitting procedure to describe variability. The GLM, introduced by Nelder and Wedderburn (1972), has been used to describe variabilities, including discrete count data. Because a normal distribution is continuous and can take negative values, it is inappropriate for count data, which are discrete and can only take zero and positive integers, such as bacterial cell numbers. A discrete probability distribution integrated into the GLM would be suitable for expressing biological count data instead of a continuous distribution such as a normal distribution. The Poisson distribution is often used to describe death events at certain time intervals in survival analyses (Dickman et al., 2004; Dickman and Coviello, 2015). A negative binomial distribution is used for over dispersal count data in the field of ecology (Ver Hoef and Boveng, 2007). The GLM can be handled by both frequentist statistics and Bayesian inference. A GLM with Bayesian inference is often used to avoid over fitting (Dey et al., 2000). A Bayesian GLM can flexibly integrate various probability distributions as model residuals and parameter uncertainty.

Another problem in the current frequentist statistical fitting procedure is the point estimation of a model parameter. The model parameters in frequentist methods, such as the least-squares method and maximum likelihood, are often estimated by fitting the model to the data. The parameters are determined at one point in the estimation. However, because the experimental data are uncertain in a real situation, the obtained parameters are also uncertain (Garre et al., 2020; van Boekel, 2020). Therefore, the estimated parameters exhibit unexplained fluctuations (van Boekel, 2020), and parameter estimation requires considering parameter errors (Dolan and Mishra, 2013). Furthermore, many types of uncertainties exist, such as model uncertainty and parameter uncertainty (FAO/WHO, 2008). In previous studies, model parameters were estimated using Bayesian inference (Jaloustre et al., 2011; Koyama et al., 2019; van Boekel, 2020). Bayesian inference has been used as a means to quantitatively estimate parameter uncertainty (Pouillot et al., 2003; Crépet et al., 2009; Koyama et al., 2019).

In the present study, a Bayesian GLM was introduced to fit observed bacterial inactivation data and growth data, and simulate bacterial behavior considering variability and uncertainty. Two types of bacteria were investigated to show applicability of the model to spoilage and pathogenic bacteria. For the inactivation data, we used datasets published in literature pertaining to the thermal inactivation of *Bacillus simplex*. As the growth data, the data obtained by investigating the growth of *Listeria monocytogenes* at 25°C were used. The data used contained three observed colony count replications for developing kinetic models, and over 50 observed colony count replications for validating bacterial behavior with small initial cell numbers. Individual cell heterogeneity and initial cell numbers were considered as variability and described using several probability distributions integrated into the model residual. The parameter uncertainty was obtained by Bayesian inference. From fitting to prediction, we consistently consider the variability in bacterial behavior. The modeling procedure considering the variability and uncertainty can contribute to improving risk-based processing design and exposure assessment.

## Materials and Methods

### Dataset

#### Inactivation Dataset

The data reported by Abe et al. (2019) were used in this study. In their study, *Bacillus simplex*, which is a psychrophilic spore-forming bacterium, originating from pasteurized milk acquired from Hokkaido Research Organization (Japan). The strain was cultured in Nutrient Agar (Eiken, Tokyo, Japan) with some components and then in Nutrient Broth (Merck) with some components at 30°C for 24 h, respectively. *Bacillus simplex* spores were obtained by culturing in Spo8-agar (Faille et al., 2007; Helmond et al., 2017). The *Bacillus simplex* in the suspension with 10^5^ cells was thermally inactivated at 94°C for kinetic evaluation. Viable counts were estimated by plating onto nutrient agar (Eiken, Tokyo, Japan) at 30°C after 2 days. Three independent trials were conducted. Furthermore, 60 replications of bacterial inactivation with an initial cell number of 10^n^ (*n* = 1–3) were used to observe the variation in bacterial inactivation.

#### Growth Dataset

##### Bacterial Strain and Inoculum Preparation

*Listeria monocytogenes* (ATCC 19118) was used in the present study. The bacteria was maintained at –80°C in tryptic soy broth (TSB; Merck, Darmstadt, Germany) containing 10 vol/vol% glycerol. The strain was activated by incubating the cells at 37°C for 24 h on tryptic soy agar (TSA; Merck) and twice at 37°C for 24 h in 5 mL of TSB to obtain a homogeneous and stable cell population. The cells were then collected by centrifugation (3,000 × *g* for 10 min). The resulting pellet was washed twice with TSB and re-suspended in 5 mL of TSB before the experiments.

##### Kinetic Evaluation of Bacterial Growth

Bacterial growth was investigated by colony counting methods. The inoculum [1 × 10^5^ colony-forming units (CFU)/mL] was prepared by series 10-fold dilutions in TSB. Aliquots (100 μL) were dispensed into the wells of 8-well polymerase chain reaction (PCR) microplates for cell concentration of 10^4^ CFU/100 μL per well. The high initial cell concentration was investigated for kinetic evaluation to avoid interference of variability derived from low cell concentrations. The microplates were incubated at 25°C. Samples were withdrawn at regular intervals to obtain kinetic data of microbial growth. At each sampling time, 8-well PCR microplates were incubated at 5°C to prevent further bacterial growth. The entire sample (100 μL) from each well was diluted by serial 10-fold dilution in TSB. The bacterial cell number was determined by plating 100 μL of the diluted suspensions on TSA plates, which were then incubated at 37°C for 24 h. The experiment was repeated independently three times.

##### Stochastic Evaluation of Bacterial Growth

Bacterial growth with a small number of initial cells (*n* = 50) were examined to evaluate the variation in cell growth. Suspensions with average of 10^n^ (*n* = −1−1) CFU/100 μL were prepared by 10-fold dilution in TSB. Aliquots (100 μL) from the same inoculum culture were dispensed into wells of an 8-well PCR microplate by using an 8-channel micropipette. Cell growth (*n* = 50 replicates) was independently assessed in 50 wells of multiple 8-well PCR microplates. The microplates were incubated at 25°C. Samples were withdrawn at regular intervals to obtain probabilistic data of microbial growth. At each sampling time, the 8-well PCR microplates were incubated at 5°C to prevent further bacterial growth. The bacterial cell numbers were determined by direct plating of 100 μL of the culture onto TSA plates without dilution (average of initial cell numbers of 0.1: examined after 0–10 h; 1: 0–6 h; and 10: 0–4 h) or after diluting (1 cells: 8 and 10 h; 10 cells: 6, 8, and 10 h). The plates were incubated at 37°C for 24 h. Fifty independent replicates were analyzed.

### Modeling

**FIGURE 1:**
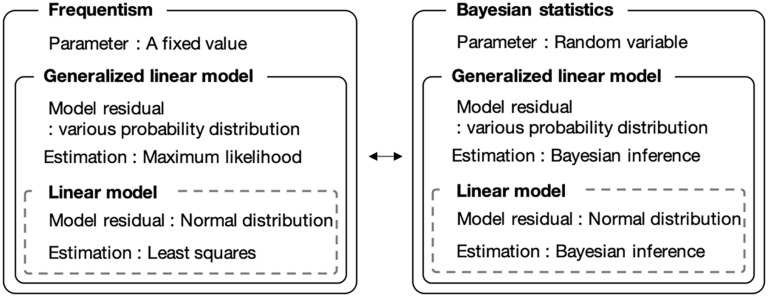
Comparison of frequentist and Bayesian statistical modeling. Bayesian statistics allows the use of parameters as random variables. The generalized linear model allows to use model residuals with various probability distributions.

We introduced a Bayesian GLM instead of the currently used least-squares method. The Bayesian GLM can flexibly integrate various probability distributions as model residuals and parameter uncertainties, unlike the least-squares method. Figure 1 shows a conceptual diagram of the fitting procedure. Because the number of bacteria is count data, we used the Poisson distribution and negative binomial distribution as model residuals instead of the normal distribution.

#### Bayesian GLM for Inactivation Dataset

**FIGURE 2:**
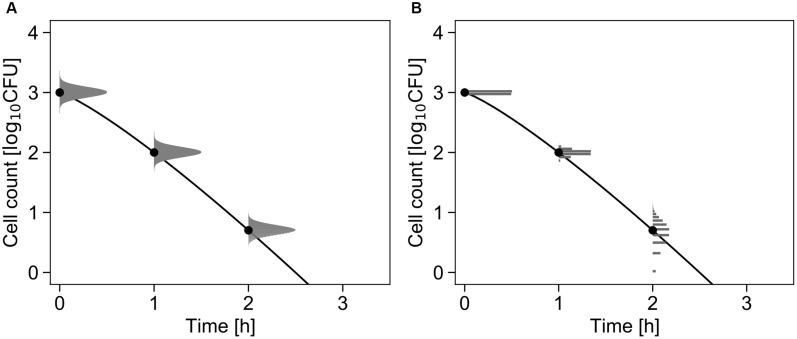
Comparison of the differences in the probability distributions assumed to represent bacterial variation during the inactivation process. Each graph shows changes in the probability density of the survival cell number with δ = 1, *p* = 1.2, and *N*_0_ = 10^3^ cells (Equations 1 and 3). The solid line and points represent the inactivation kinetic and mean value of the probability distribution, respectively. The logarithmic survival cell number is assumed to follow a normal distribution **(A)**, whereas the survival cell number is assumed to follow a Poisson distribution **(B)**.

Figure 2A shows the conceptual diagram of the inactivation model. In the least-squares method used in frequentist statistics, the error in the logarithmic number of cells is assumed to follow a normal distribution (Figure 2A). In contrast, the error is assumed to follow a Poisson distribution in the inactivation process when using the GLM (Figure 2B).

First, we show a kinetic model based on the least-squares method. The data with 10^5^ inactivated cells were fitted to the Weibull model. The Weibull model was fitted to the inactivation data, and the Weibull model is described in Equation (1):

(1) $$l\,o\,g_{10}\,\frac{N_{t}}{N_{0}}=-{\left(\frac{t}{\delta }\right)}^{p}$$

where *t*, *N_t*, *N_0*, *p*, and δ denote the inactivation time, bacterial population at time *t*, initial number of cells, shape parameter, and scale parameter, respectively. Curve regression to the Weibull model was conducted using a non-linear least-squares method.

Next, we construct the GLM. Equation (1) can be transformed into Equation (2):

(2) $$N_{t}=N_{0}\times 10^{-{\left(t/\delta \right)}^{p}}$$

Here, the bacterial cell number experimentally obtained via a dilution series was assumed to follow a Poisson distribution (Koyama et al., 2016). Therefore, it can be assumed that the initial cell data follow a Poisson distribution. In addition, we assumed that the inactivation rate of each cell was equal and that a cell inactivation event was independent of another event. The number of surviving cells can also follow a Poisson distribution under the assumption that the initial cells (which follow the Poisson distribution) die at random (Aguirre et al., 2009). Therefore, it can be assumed that the observed values of the number of surviving bacteria obtained at each time are taken from a Poisson distribution with an average of *N*_0_×10^−(*t*/δ)*p*^, and the bacterial population at time *t* (*N_t*) can be described as in Equation (3):

(3) $$N_{t}∼P\,o\,i\,s\,s\,o\,n\,\left(N_{0}\times 10^{-{\left(t/\delta \right)}^{p}}\right)$$

In this study, the parameters (δ and *p*) and the initial bacterial count (*N_0*) were estimated from the heating time (*t*) and the number of surviving bacteria at each time (*N_t*). The random variable in the number of cells is Poisson-distributed, which is equivalent to the model residual of the dependent variable in the GLM. The Bayesian GLM is constructed using Equation (3).

#### Bayesian GLM for the Growth Dataset

**FIGURE 3:**
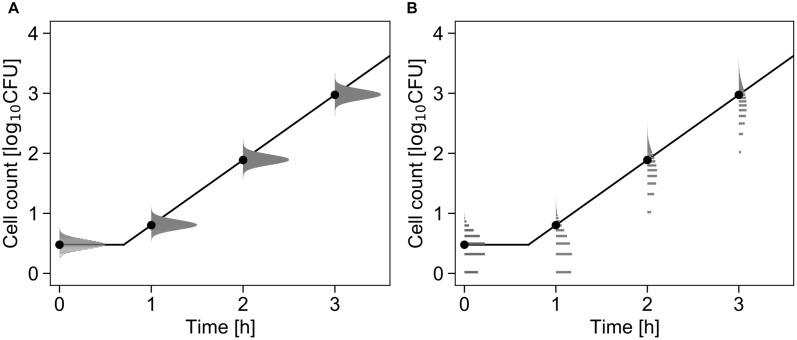
Comparison of the differences in the probability distributions assumed to represent bacterial variation during the growth process. Each graph shows changes in the probability density of the cell number with μ = 2.5, λ = 0.7, and *N*_0_ =  3 cells (Equations 4 and 5). The solid line and points represent the growth kinetics and mean value of the probability distribution, respectively. The logarithmic cell number is assumed to follow a normal distribution at all times **(A)**. The initial cell number is assumed to follow a Poisson distribution, and the growth number of divisions is assumed to follow a negative binomial distribution **(B)**.

Figure 3 shows a conceptual diagram of the growth model. In the least-squares method used in frequentist statistics, the error in the logarithmic number of cells is assumed to follow a normal distribution (Figure 3A). In contrast, the error in the initial cell number is assumed to follow a Poisson distribution, and the error in the number of divisions during the exponential phase is assumed to follow a negative binomial distribution (Figure 3B).

First, we show a kinetic model based on the least-squares method. The growth model used in this study was based on a three-phase linear model (Buchanan et al., 1997; McKellar and Lu, 2003) without a stationary phase. In this study, to simplify the calculation, the stationary phase was not included in the data used for the analysis. The kinetic model is described in Equation (4):

(4) $$\log_{10}N_{t}=\log_{10}\left(N_{0}\times exp\left(\mu \times \left(t-\lambda \right)\right)\right)\left(t>\lambda \right)$$

$$\log_{10}N_{t}=\log_{10}N_{0}\left(t\leq \lambda \right)$$

where *t*, *N_0*, *N_t*, μ, and λ denote the incubation time, initial cell number, number of bacteria, maximum growth rate, and lag time, respectively. Curve regression to the growth data was conducted using the least-squares method, and μ and λ were estimated.

Next, we construct the GLM. Here, as with the inactivation model, since the initial cells were experimentally obtained using a dilution series, the initial cells were assumed to follow a Poisson distribution. In addition, we assumed that the exponential growth rate of the individual cells is equal and that cell division is independent of another event (Coleman and Marks, 1999). Under these assumptions, a pure birth process is used to calculate the stochastic growth of bacteria (Renshaw, 1993; Coleman and Marks, 1999). In the pure birth process, the number of divisions can be described as a negative binomial distribution, as in Equation (5):

(5) $$D_{t}∼Negbin\left(N_{0},exp\left(-\mu \times \left(t-\lambda \right)\right)\right)\left(t>\lambda \right)$$

$$N_{0}∼P\,o\,i\,s\,s\,o\,n\,\left(N_{0}\right)$$

$$D_{t}=0\left(t\leq \lambda \right)$$

$$N_{t}=N_{0}+D_{t}$$

where *t*, *D_t*, *N_0*, *N_t*, μ, and λ denote the incubation time, a total number of cell divisions in bacterial population up to time *t*, number of initial cells, number of bacteria, maximum growth rate, and lag time, respectively. In this study, Bayesian inference was conducted using the growth dataset in both the lag and exponential phases. The parameters (μ and λ) were estimated from the incubation time (*t*) and the number of bacteria at each time point (*N_t*). The Bayesian GLM is constructed using Equation (5).

#### Computation

In this study, the parameters were estimated using Bayesian inference. In Bayesian inference, the obtained data were considered to have been generated from a probability distribution, and all the parameters were estimated as a probability distribution. Bayesian inference can combine priors, even if no prior information is available. In this study, we used a uniform distribution as a non-informative prior distribution because there was no prior information. For each model, inferences were made on 10^4^ iterations with four independent chains. The first 5,000 iterations of 10^4^ iterations were removed as a warm up period and the rest 5,000 iterations were used as posterior parameters estimation. Convergence was verified by both visually checking the Markov Chain Monte Carlo chain traces and examining the Gelman and Rubin diagnostic called R-hat. The R-hat value should be close to 1.0. Computations were performed using PyStan and Python (version 3.7.7).

### Simulation

#### Inactivation Dataset

Two parameters (δ and *p*) were obtained in pairs, and 2 × 10^4^ sets (5,000 iterations × 4 chains) were obtained by conducting Bayesian inference. We simulated the inactivation behavior with average of initial cell numbers of 10^n^ (*n* = 1–3) using 2 × 10^4^ sets of parameters. We assumed that the initial cell number and the survival cell number at each time followed a Poisson distribution. The time *t* (min) was set from 0 to 6 at 0.05 (min) intervals. The time and parameter values were substituted into Equation (2), and the survival cell numbers (*N_t*) were calculated corresponding to each time and each parameter. We generated random numbers as many as 2 × 10^4^ sets from the Poisson distribution with mean *N*_0_×10^−(*t*/δ)*p*^. The number obtained here was defined as the number of surviving cells at that time. The 2 × 10^4^ predicted results were arranged in an ascending order, and the points corresponding to the top 2.5% and the bottom 2.5% were plotted. These lines were set as the 95% predicted interval, and the predicted results were compared with the observed values for 10^n^ (*n* = 1–3) cell inactivation. The procedure for evaluating the predicted results was mostly based on a previous study (Hiura et al., 2020). The 2 × 10^4^ prediction results at each time points were arranged in ascending order. If observed colony count was greater than the prediction corresponding to the lower 2.5% and less than the prediction corresponding to the upper 2.5%, the observed colony count was considered to be within the prediction range. The ratio of the number in the 95% prediction interval among the 60 observed values was calculated as the accuracy.

#### Growth Dataset

The parameters obtained in section “Computation” were used to predict the growth behavior with average of initial cell numbers of 10^n^ (*n* = −1−1). We predicted the growth behavior at 10^n^ (*n* = −1−1) initial cells using 2 × 10^4^ sets of parameters.

(1) Simulation of initial bacterial number

The initial number of bacteria was assumed to follow a Poisson distribution. We generated random numbers as many as 2 × 10^4^ sets from the Poisson distribution with mean *N_0*. The number obtained here was set as the number of initial cells.

(2) Simulation of the growth number of cells

The estimated parameters (μ and λ), the initial number of bacteria, and the time (*t_i*) to predict the number of bacteria were substituted into Equation (5), and the random number following a negative binomial distribution was generated. At the desired time (*t_i*), if *t*_*i*_≤ λ, the bacterial population could not grow. When *t*_*i*_ > λ, we generated a random number following a negative binomial distribution. The obtained value was the division number at each time. The number of bacteria at each time point was predicted by adding this division number and the initial cell number.

The 2 × 10^4^ simulated results obtained by the above procedure were arranged in an ascending order, and the points corresponding to the top 2.5% and the bottom 2.5% were plotted at each time. These lines were set at 95% predicted intervals, and the simulated results were compared with the observed values for 10^n^ (*n* = −1−1) cell growth behavior. The procedure for evaluating the predicted results was as with section “Inactivation Dataset.” The ratio of the number in the 95% prediction interval among the 50 observed values was calculated as the accuracy.

## Results

### Bayesian Inference and Prediction of Bacterial Behavior in the Inactivation Process

**FIGURE 4:**
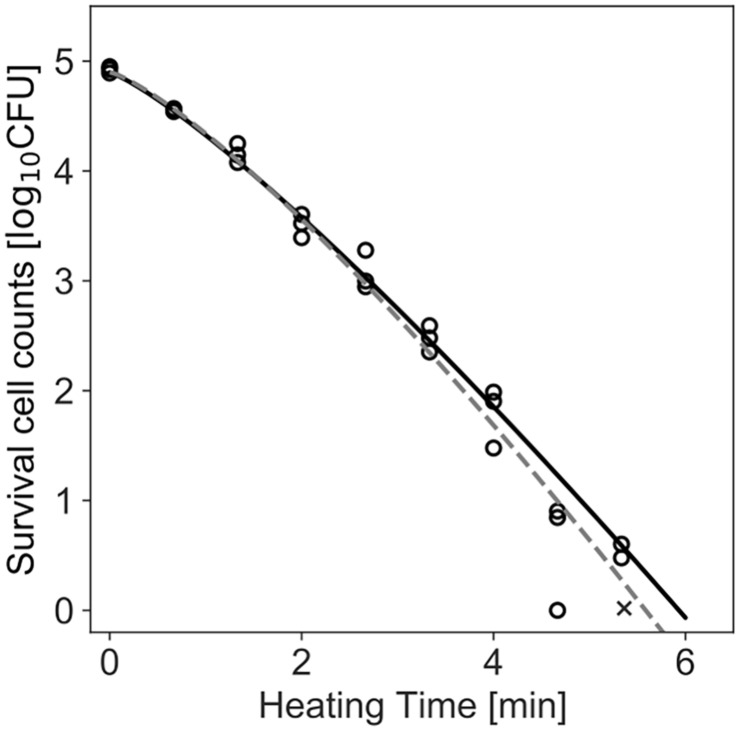
Survival kinetics of *Bacillus simplex* with a population of 10^5^ cells, heated at 94°C. Each time has three replications. No colonies are detected at the time indicated by a cross (×). The dashed line indicates the fitted Weibull model by the least-squares method. The solid line indicates the median of the fitting by Bayesian inference.

**FIGURE 5:**
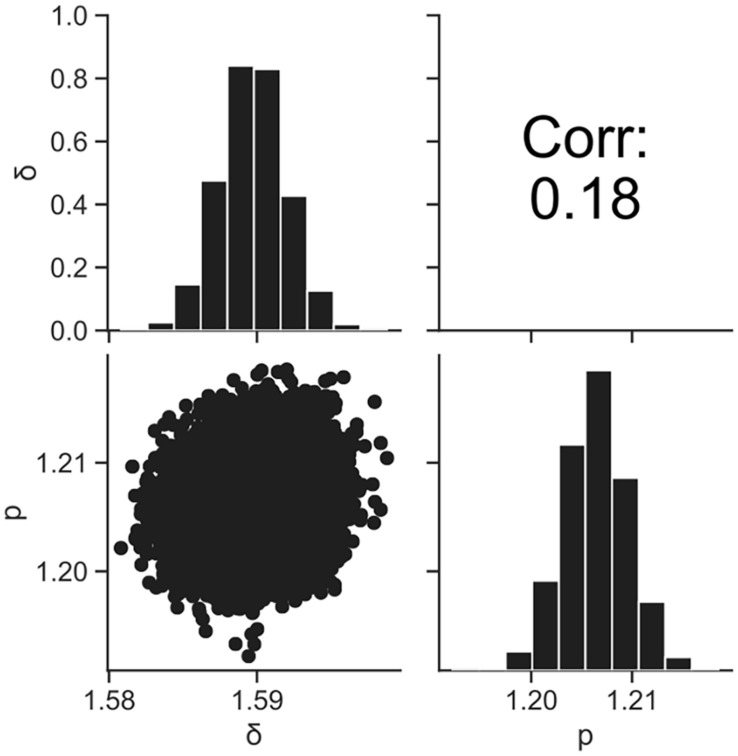
Histograms (on the diagonal), correlation plots (under the diagonal), and correlation coefficients (over the diagonal) of the estimated parameters δ and *p* resulting from Bayesian inference.

Figure 4 shows the dataset of the inactivation of *B. simplex* with 10^5^ cells, and the fitted results by the least-squares method and Bayesian inference. Both kinetic and Bayesian fitting yielded similar results. As a regression to the Weibull model using the least-squares method in frequentist statistics, δ and *p* were 1.58 (standard deviation was 0.12) and 1.26 (standard deviation was 0.09), respectively. The root-mean-square error as a goodness-of-fit index was 0.18, which indicates a good fit. Figure 5 shows the posterior distributions of the parameters δ and *p* with Bayesian inference. Bayesian inference was conducted using data with a survival cell count of 0 colony forming unit (CFU). The R-hat value was 1.0 for each parameter, which indicates a good convergence. The mean values of δ and *p* were 1.59 (standard deviation was 0.11) and 1.21 (standard deviation was 0.15), respectively. The correlation coefficient between parameters δ and *p* was 0.18, indicating a poor positive correlation. The average values of the parameters estimated by Bayesian inference were comparable to the results estimated by the least-squares method used in frequentist statistics. However, the parameters were narrowed down to one point in the least-squares method, whereas the parameters were estimated as probability distributions in Bayesian inference.

**FIGURE 6:**
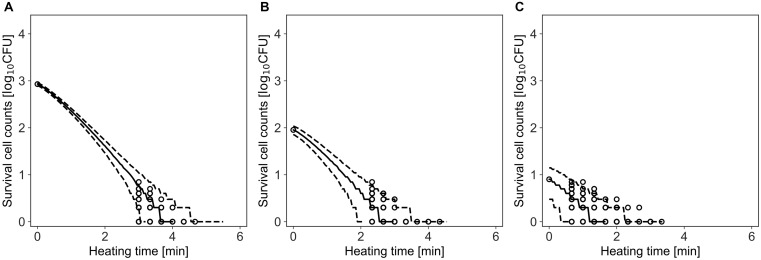
Comparison between observed and simulated Inactivation of *Bacillus simplex* with initial cell numbers of 10^3^ **(A)**, 10^2^ **(B)**, and 10 cells **(C)**. The solid and dashed lines indicate the median of the prediction and the 95% predicted interval, respectively. Observed data are indicated by a circle.

Figure 6 shows a comparison between the observed data and the simulated results by the model. The rates of validity of the number of cells within the 95% prediction band were 96, 99, and 96% for initial cell numbers of 850, 90, and 8, respectively. This result indicates that the simulation by this model covers almost the entire variation in the inactivation behavior.

### Bayesian Inference and Simulation of Bacterial Behavior in the Growth Process

**FIGURE 7:**
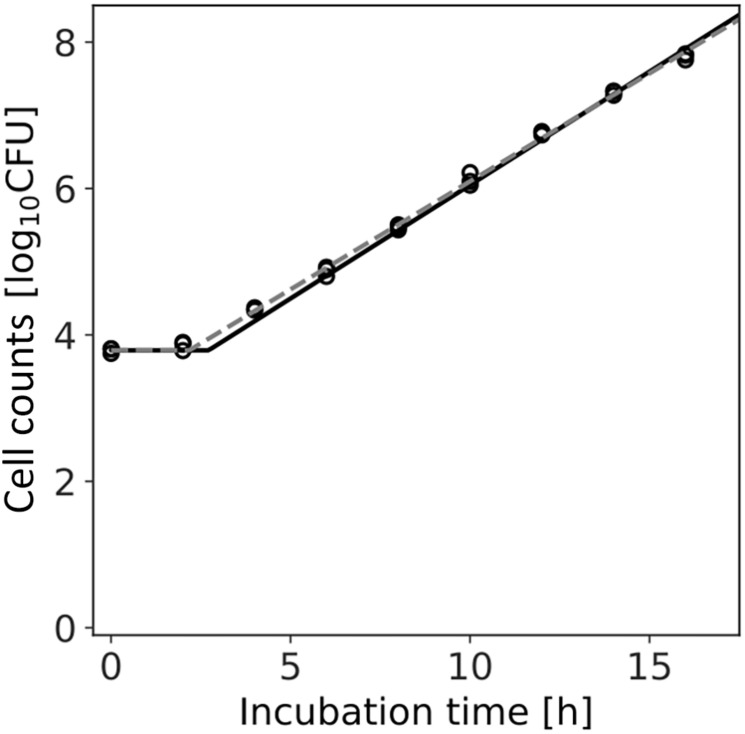
Growth kinetics of *Listeria monocytogenes* with a population of 10^4^ cells incubated at 25°C. Each time has three replications. The dashed line indicates the growth kinetics fitted by the least-squares method used in frequentist statistics. The solid line indicates the median of the fitting by Bayesian inference.

**FIGURE 8:**
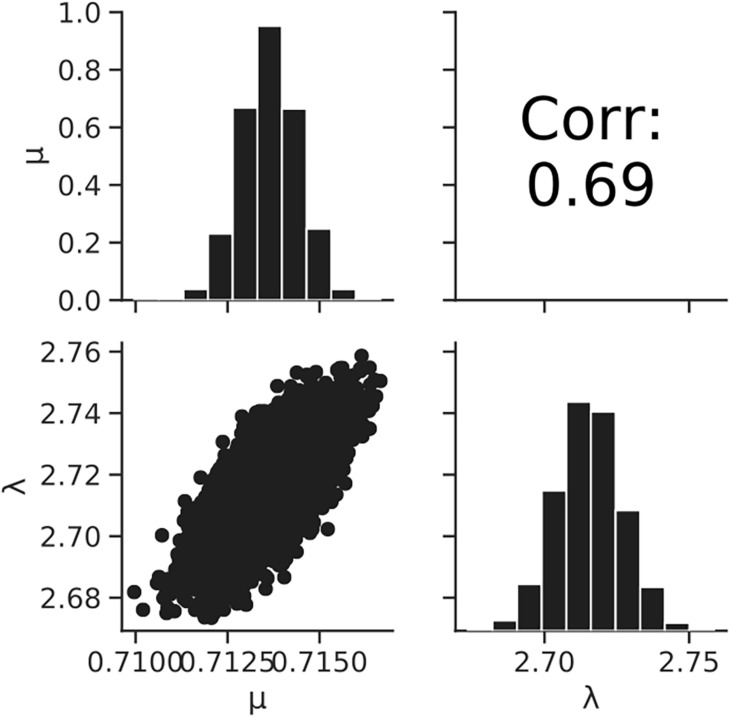
Histograms (on the diagonal), correlation plots (under the diagonal), and correlation coefficients (over the diagonal) of the estimated parameters μ and λ resulting from Bayesian inference.

Figure 7 shows the dataset of the growth behavior of *L. monocytogenes* with a population of 10^4^ cells incubated at 25°C, and the fitted results by the least-squares method and Bayesian inference. Both kinetic and Bayesian fitting yielded similar results. As a regression to the kinetic model using the least-squares method, μ and λ were 0.68 (standard deviation was 0.01) and 2.18 (standard deviation was 0.15), respectively. The RMSE was 0.05. Figure 8 shows the posterior distributions of the parameters μ and λ with Bayesian inference. The R-hat value was 1.0 for each parameter, which indicates a good convergence. The mean values of μ and λ were 0.71 (standard deviation was 0.14) and 2.72 (standard deviation was 0.01), respectively. The correlation coefficient between the parameters μ and λ was 0.69, indicating a positive correlation. The average values of the parameters estimated by Bayesian inference were comparable to the results estimated by the least-squares method used in frequentist statistics.

**FIGURE 9:**
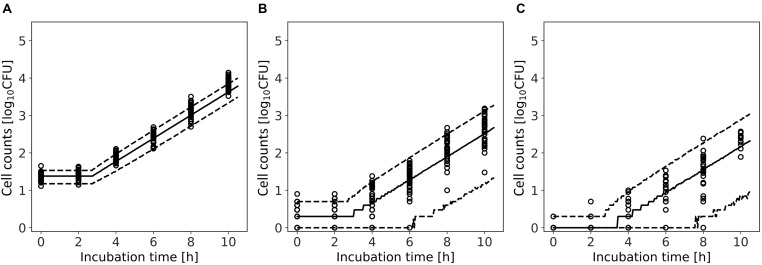
Comparison between observed and simulated growths of *Listeria monocytogenes* with average of initial cell numbers of 10 **(A)**, 1 **(B)**, and 10^–1^ cell **(C)**. The solid and dashed lines indicate the median of the simulation and the 95% predicted interval, respectively. Observed data are indicated by a circle.

Figure 9 shows a comparison between the observed data and the results simulated by the model. The rates of validity of the number of cells within the 95% predicted interval were 80, 94, and 96% for average of initial cell numbers of 24, 2, and 0.3 cell, respectively. For 0.3 and 2 cell, the accuracy was calculated for observed values greater than 0 CFU. This result indicates that the model simulation covers almost the entire variation in the growth behavior of the small initial cell number.

## Discussion

In the present study, we introduced Bayesian GLM to incorporate the variability and uncertainty into a predictive model. The estimated results of the parameter uncertainty by both the inactivation and growth models were represented in the form of a probability distribution (Figures 5, 8), which has not been considered in the conventional least-squares method. The estimated parameters enabled to predict the inactivation behavior at various initial cell numbers, such as 10^3^, 10^2^, and 10 cells, with an accuracy of over 90% (Figure 6). The estimated parameters of the growth model enabled to predict the growth behavior at various initial cell numbers, such as 24, 2, and 0.3 cell (Figure 9). In particular, for less than 2 cells, the growth behavior was predicted with a high accuracy of over 90%. The present model expresses the variation in the bacterial behavior at low cell concentrations, which is remarkable given the individual cell heterogeneity (Koutsoumanis and Lianou, 2013; Aspridou and Koutsoumanis, 2015). The Bayesian GLM was able to fit the inactivation and growth of the bacterial population and predict the bacterial behavior considering variability and uncertainty. This modeling procedure allows to consistently consider the variations in the actual bacterial behavior, from fitting to prediction.

As a means of expressing the variation in bacterial behavior, a stochastic model has been developed, that expresses the variability in bacterial behavior with a probability distribution (FAO/WHO, 2008). Several models that can represent variability in bacterial behavior have been developed. Previous studies have clarified that variability due to individual cell heterogeneity can be expressed using a probability distribution and Monte Carlo simulation (Poschet, 2003; Aspridou and Koutsoumanis, 2015). Others have suggested combining kinetic models with computer simulations to demonstrate variability in bacterial behavior (Abe et al., 2019; Hiura et al., 2020). Even if the variability is expressed by a Monte Carlo simulation after fitting the model, such a method is mathematically inappropriate because there are discrepancies in the model residuals during and after fitting. Therefore, in the present study, a consistent procedure, from fitting to prediction, was implemented by introducing the GLM and model fitting to the data considering the variability due to individual cell heterogeneity. The model fitting to the data and the bacterial behavior simulation can be conducted under the assumption of the same probability distribution in the process of fitting the model to the data and the simulation (Equations 3 and 5). It is reasonable to consider variability and uncertainty while fitting to the data instead of doing so after the fitting.

In the least-squares method, the logarithmic number of bacterial populations is treated as a continuous number because a normal distribution is used as the error distribution. The cell count is logarithmically analyzed in data analyses in the field of microbiology. Because the logarithm cannot be taken for 0 CFU, the count of 0 CFU is omitted from the dataset. O’Hara and Kotze (2010) suggested that distributions designed to deal with counts, such as the Poisson distribution or negative binomial distribution, should be used to fit count data instead of using a continuous distribution such as a normal distribution. O’Hara and Kotze (2010) also insisted that a log-transformation of count data should be considered when dealing with zero observations. With a discrete distribution, it is possible to make fitting and predictions, including for data with a cell number of 0 CFU. Therefore, in the present study, the number of bacteria was treated as a discrete number when fitting using the GLM (Equations 3 and 5). We expressed the variability in the bacterial cell number by introducing a discrete distribution, i.e., a Poisson distribution (Equations 3 and 5), and a negative binomial distribution (Equation 5). We were able to use data with a survival cell count of 0 CFU for parameter estimation (Figure 4) and prediction, since the Poisson distribution was used as the error distribution of the cell count. With the use of discrete distributions, the data of bacterial counts, including 0 CFU, can be used for analyses. Thus, data loss during analysis can be prevented. A discrete probability distribution is useful for expressing the number of bacteria counts, particularly in the case of a low dose, including zero.

Variability and uncertainty simultaneously appear experimentally (Poschet, 2003; Park and Lee, 2008). It is necessary to consider both these factors when conducting exposure assessments (FAO/WHO, 2008). Bacterial behavior is characterized by variability and uncertainty, and the need to consider both has been pointed out (Nauta, 2000). It is relatively easier to define variability using an equation than uncertainty, since variability is derived from some exact factors such as individual cell inactivation time as individual cell heterogeneity (Aspridou and Koutsoumanis, 2015). Therefore, the definition of variability such as individual cell inactivation time and between-strains is an important first step, since understanding variability can help determine the degree of uncertainty. The better we know the variability, the clearer the uncertainty. We defined the variability in the number of bacteria during the inactivation and growth processes using the Poisson distribution and negative binomial distribution (Equations 3 and 5), which can be a fundamental assumption for the further analysis of the variability and uncertainty in bacterial behavior.

Some other distributions such as Poisson-lognormal and Poisson-gamma distributions were used to describe number of cells in food production, where many factors affect the heterogeneity of microbial numbers among food units (Gonzales-Barron and Butler, 2011). Poisson-lognormal and Poisson-gamma distributions are used to describe over dispersion of count data (Congdon, 2006). These probability distributions may be possible choice to predict bacterial population behavior in food.

Only colony count data have been used for constructing kinetic models in Bayesian GLM that considers variability and uncertainty in bacterial behavior. Colony count data can be found not only in literature but also in databases such as ComBase^1^. So far, risk related to food borne pathogens has been assessed using these accumulated data. The model proposed in the present study can help represent the variability and uncertainty in bacterial behavior using existing published data, providing a more realistic quantitative exposure assessment compared to using the conventional least-squares method. The proposed modeling procedure can help account for the variability and uncertainty in risk-based modeling.

## Conclusion

The present study illustrated the construction of a Bayesian GLM considering the variability and uncertainty in bacterial inactivation and growth behavior. This modeling procedure allowed to consistently assume a probability distribution representing the variation in bacterial behavior throughout the fitting process for simulating bacterial behavior. The developed models enable a more explicit illustration of the variation in bacterial behavior via probability distributions, because the models are based on probabilistic theory. For example, the variation in bacterial numbers following a Poisson distribution was derived from experimentally prepared bacterial cells via a dilution process. In addition, the probability distributions of the growth or inactivation processes were assumed to be independent of other biological events. Thus, the models developed in the present study provide a reliable foundation for representing the variability and uncertainty. The Bayesian GLM can separately describe the variability and uncertainty, which cannot be done using the conventional least-squares methods used in frequentist statistics.

## Data Availability Statement

The datasets and codes in this article can be found online at: https://github.com/Satoko-Hiura/Bayesian-generalized-linear-model-for-simulating-bacterial-inactivation-growth.git.

## Author Contributions

SH, HA, KK, and SK designed the study and wrote the manuscript. SH performed the study and analyzed the data. All authors contributed to the article and approved the submitted version.

## Conflict of Interest

The authors declare that the research was conducted in the absence of any commercial or financial relationships that could be construed as a potential conflict of interest.

## Footnotes

1 www.combase.cc
